# Microbiological and Molecular Diagnosis of Mucormycosis: From Old to New

**DOI:** 10.3390/microorganisms9071518

**Published:** 2021-07-16

**Authors:** Nina Lackner, Wilfried Posch, Cornelia Lass-Flörl

**Affiliations:** Department of Hygiene and Medical Microbiology, Medical University Innsbruck, 6020 Innsbruck, Austria; nina.lackner@i-med.ac.at (N.L.); wilfried.posch@i-med.ac.at (W.P.)

**Keywords:** Mucorales, diagnosis, DNA, PCR, specificity, sensitivity, clinical evaluation, standardization, culture, microscopy

## Abstract

Members of the order Mucorales may cause severe invasive fungal infections (mucormycosis) in immune-compromised and otherwise ill patients. Diagnosis of Mucorales infections and discrimination from other filamentous fungi are crucial for correct management. Here, we present an overview of current state-of-the-art mucormycosis diagnoses, with a focus on recent developments in the molecular field. Classical diagnostic methods comprise histology/microscopy as well as culture and are still the gold standard. Newer molecular methods are evolving quickly and display great potential in early diagnosis, although standardization is still missing. Among them, quantitative PCR assays with or without melt curve analysis are most widely used to detect fungal DNA in clinical samples. Depending on the respective assay, sequencing of the resulting PCR product can be necessary for genus or even species identification. Further, DNA-based methods include microarrays and PCR-ESI-MS. However, general laboratory standards are still in development, meaning that molecular methods are currently limited to add-on analytics to culture and microscopy.

## 1. Introduction

Diagnosing invasive mucormycosis is challenging as clinical symptoms and imaging are non-specific, blood cultures are commonly negative and specific biomarkers are not available [1]. Conventional methods such as microscopy and culture remain the gold standard but may lack sensitivity, in addition, cultures may take a long time to yield a positive result [2]. In this context, the development of alternative culture-independent methods, which are based on the detection of genetic material, has been pursued by many researchers in the last decades. This review aims to give a comprehensive overview of current investigational and commercial molecular assays and to discuss the potential, limitations, and perspectives in this rapidly evolving field.

## 2. Histological and Microscopic Diagnosis

Microscopy of primarily sterile body specimens is an important tool in the diagnosis of invasive fungal infections, as positivity provides proof of an infection. Bronchoalveolar-lavage fluids and sinus tissue must be handled as sterile specimens [3]. Microscopy supports a wide and easy application as well as a rapid processing time. Sensitivity depends on the source and quality of specimens obtained; Mucorales hyphae are vulnerable and therefore may be hurt when preparing the sample (grind), which in turn may result in decreased fungal growth.

**Figure 1:**
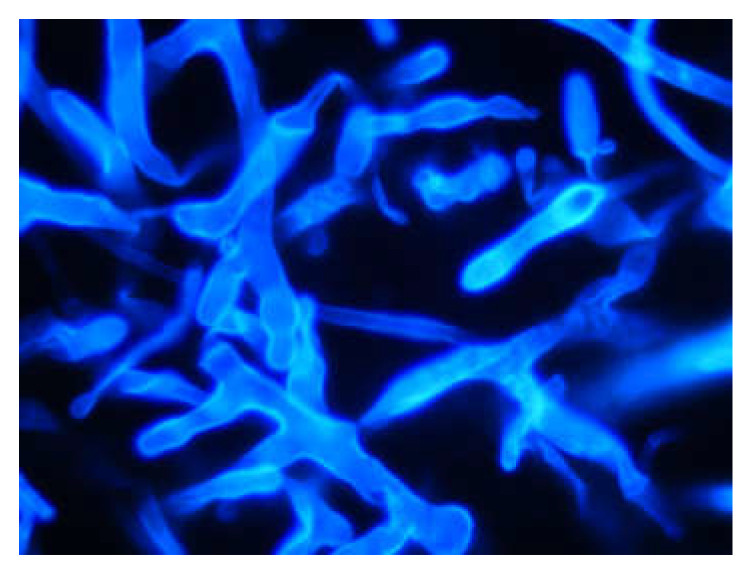
Broad, ribbon-like, non-septate hyphae of *Mucor* sp. with wide-angle branching, stained with fluorescent brightener (Calcofluor White × 400).

Direct examination applying optical brighteners (Blankophor, Calcofluor White) supports a quick and presumptive diagnosis of mucormycosis [4]. Mucorales hyphae are variable in broadness (6 to 25 µm), non- or sparsely-septated, have irregular branching (ribbon-like), and angles variable up to 90° (wide-angle bifurcations) (Figure 1) [2,4]. In addition, standards of care are Gomori’s methenamine silver or periodic acid–Schiff stain. Tissue histopathology displays a neutrophilic or granulomatous inflammation, the latter characteristic is absent in immunosuppressed patients [4]. Invasive mucormycosis include prominent infarcts and angioinvasion; neutropenic patients present substantial angioinvasion in contrast to non-neutropenic patients [4]. In most cases, microscopy prohibits conclusive differentiation of Mucorales from *Aspergillus* and other filamentous fungi [5]; special occasions (clear fungal morphology) may provide initial information on the fungal class. The determination between septate (e.g., Aspergillus) and non-septate hyphae (e.g., Mucorales) is of clinical significance, as this strongly affects antifungal treatment [4].

## 3. Microbiological Diagnosis

Culture is the gold standard for fungal specification and, in addition, enables antifungal susceptibility testing [1]. Mucorales are fast-growing (3–5 days) on standard fungal culture media (e.g., Sabouraud agar and potato dextrose agar) incubated at 25 °C to 30 °C. It is important to note that, even in positive microscopy, only 50% of cases are culture positive [2]. Based on the lack of clinical breakpoints, the implementation of antifungal susceptibility testing in the management of mucormycosis is not supported [4].

The identification of culture isolates by matrix-assisted laser desorption ionization time-of-flight mass spectrometry (MALDI-TOF) is a quick and reliable method for bacterial and yeast infections. In contrast, the handling of filamentous fungi is more complicated, as multiple pre-analytic steps are needed and the age of the culture influences the resulting spectra [6]. Nevertheless, methods for MALDI-TOF identification of Mucorales isolates were evaluated successfully by several authors [6,7,8,9]. Schwarz et al. (2019) studied 38 Mucorales isolates, covering 12 different species, which previously were identified by molecular-based methods [6]. A database containing 10 main spectra profiles was created and resulted in good interspecies discrimination; database accuracy resulted in a log-score > 2. If enough reference isolates for each species are included, identification at the species level seems possible. Shao et al. (2018) suggest improving the existing Brucker library, due to the lack of some fungal species [7]. Zvezdanova et al. (2019) adopted the method for filamentous fungi by applying a mechanical lysis followed by a protein extraction step [9]. The implementation of an in-house library enabled accurate species (91.3%) and genus (8.7%) identification.

## 4. DNA-Based Diagnosis

The molecular detection of Mucorales is currently restricted to the detection of DNA, as ß-D-glucan and galactomannan assays do not detect this fungal group [10]. Initially, the detection of Mucorales DNA from clinical specimens was mainly developed for tissue samples as an add-on to histopathological and microbiological diagnosis [11,12]. Later, assays for blood and even urine were developed [13,14]. Compared to invasive aspergillosis, the load of circulating Mucorales DNA in serum was found to be very high, probably due to the angioinvasive nature of Mucorales infections [13,15]. Therefore, blood samples are suitable for suspicion-independent screenings of high-risk patients and therapeutic monitoring [13].

### 4.1. DNA Extractability and Biological Specifics of Mucorales

The sensitivity of DNA-based diagnostic methods is strongly influenced by the amount and extractability of fungal DNA in a clinical sample, which in turn depends on the sample type. In comparison with fresh tissue, DNA extractability from formalin-fixed paraffin-embedded tissue (FF-PET) is reduced due to the adverse effect of formalin on DNA [16]. The partial fragmentation of DNA by formalin reduces the sensitivity of the detection via PCR [4]. This can be counteracted using very short PCR amplicons [16]. Concerning the DNA extraction method, Muñoz-Cadavid et al. (2010) tested five different commercially available extraction kits and found differences in the proportion of FF-PET samples, from which DNA could be extracted and subsequently amplified in a pan-fungal PCR. [17]. Further, Scharf et al. (2020) observed that for *Aspergillus*, bead beating could significantly increase the amount of DNA extracted from serum and respiratory samples [18]. However, an inter-laboratory study displayed no significant effect of the method of extraction of Mucorales DNA from the serum on the diagnostic sensitivity [19].

Further, the characteristics and physiological status of the causative pathogen are highly relevant to the amount of DNA. As mentioned above, Mucorales hyphae are not or scarcely septated, multinucleic, and fragile compared with *Aspergillus* hyphae. For *Aspergillus oryzae,* it could be demonstrated that the number of nuclei depends on the nutrition and proliferation status of the hyphae section, and this is regulated by macroautophagy of whole nuclei as a nutrient recycling process [20,21]. Therefore, the vitality of the fungus may have an impact on the sensitivity of the diagnostic methods [22]. Further, the copy number of the target gene, mostly ribosomal DNA, is relevant for the detectability of an organism. Maicas et al. (2000) and Millon et al. (2016) estimated that the genomes of *Mucor miehei* and *Lichtheimia corymbifera* contain 100 and 25−41 copies of the ribosomal DNA unit, respectively [13,23], which is in the normal range for filamentous fungi [24,25].

### 4.2. PCR-Based Methods

**Figure 2:**
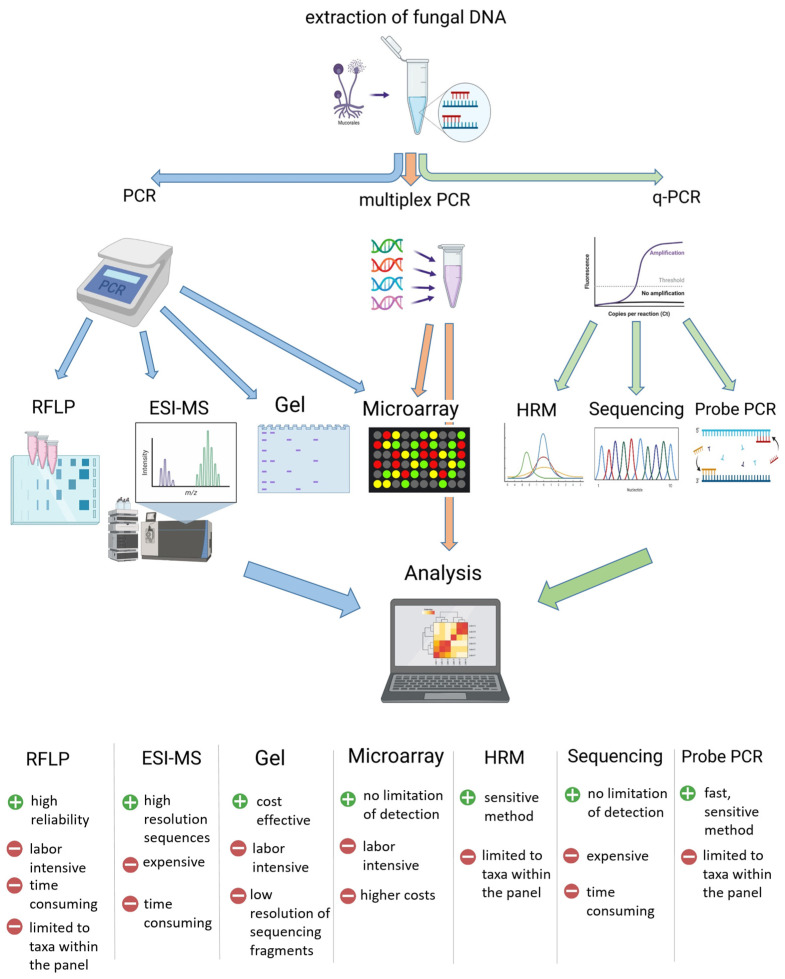
Overview of major molecular methods and their advantages and disadvantages.

Most DNA-based detection methods (except for fluorescence in-situ hybridization) apply the amplification of the target genomic information via PCR, leading to high analytic sensitivities. This high sensitivity, however, also leads to an increased risk of contamination with ubiquitous environmental fungi [26]. Target DNA can be detected during (quantitative real-time PCR) or after PCR (gel electrophoresis, microarray, and electrospray-ionization mass spectrometry (ESI-MS) sequencing) (Figure 2). The use of probes or high-resolution melt (HRM) analysis in qPCR, as well as the use of microarrays or ESI-MS, increases the specificity of the assays and reduces the false positivity rate [27]. In terms of feasibility, the need for special technical equipment and, therefore, the costs for implementation, are lower in PCR or qPCR methods compared with microarrays and ESI-MS, sequencing is not necessary or can be easily outsourced.

PCR/ESI-MS involves a PCR with multiple pairs of broad-range primers coupled with electro-spray ionization mass spectrometry [28]. ESI-MS yields a molecular fingerprint, which can be used to identify organisms by comparison with a reference database [28]. Massire et al. (2013) evaluated a PCR/ESI-MS assay for clinical isolates and found good results for *Aspergillus* and *Candida*, and moderate data for Mucorales (Table 1) [28]. Microarrays for fungal pathogens including Mucorales were developed by Spiess et al. (2007) and Hsiao et al. (2005) for 14 (including 2 Mucorales) and 64 (including 4 Mucorales) fungal taxa, respectively (Table 1) [29,30]. The assay of Spiess et al. (2007) was also evaluated for clinical specimens and yielded a sensitivity of 64% and a specificity of 80% for non-*Aspergillus* invasive fungal infections [31].

In general, PCR assays can be classified based on the specificity of their applied primers and probes (Table 1). In general, there are species/genus-specific, order (Mucorales)-specific, and pan-fungal PCR assays. Although identification of the causative Mucorales at the genus or species level has little implication for the choice of antifungal treatment, it is crucial for epidemiology [4]. Therefore, the “One World One Guideline” initiative of the European Confederation of Medical Mycology recommends the molecular identification of clinical Mucorales isolates [4].

### 4.3. Genus- to Species-Specific PCR Assays

Species/genus-specific PCR assays combine the detection and identification of the infectious fungus in one step. However, these assays are limited to the detection of taxa within their panel and therefore multiple parallel PCR assays are necessary to cover the range of possible invasive fungi. The application of multiplex qPCRs increases the feasibility in this context. Bernal-Martínez et al., (2013) presented a triplex qPCR assay for the detection of *Rhizopus orzyae*, *Rhizopus microsporus*, and *Mucor* sp. (Table 1) [41]. Salehi et al., (2016) developed two multiplex assays, which cover seven common Mucorales species in total [40]. Further, the United States Environmental Protection Agency (US EPA) has published a panel of 36 primer sets for Mold Specific Quantitative PCR to assess home mold burden [37]. This panel includes three primer sets for the genera *Lichtheimia*, *Rhizomucor*, and *Mucor*/*Rhizopus*, which were evaluated for diagnostic application multiple times (Table 2). The EPA assay can be expanded with a primer set for *Cunninghamella* spp., designed by Bellanger et al., (2018) [38].

### 4.4. Mucorales-Specific PCR Assays

In contrast, the use of Mucorales-specific primers gives a broader detection range but increases the need for additional analysis to identify the infectious agent. These post-PCR analyses can include sequencing of the amplicons, restriction-fragment-length polymorphism, microarray, and the analysis of HRM in qPCR applications (Figure 2). In this context, the most applied and modified PCR assay is from Bialek et al., (2005) (Table 1 and Table 2) [11]. In their original publication, they presented a semi-nested PCR targeting the 18S rRNA gene, which could be combined with sequencing of the amplicons to reach an identification at the genus level [11]. This assay was later used for subsequent restriction fragment length polymorphism analysis [31] and adapted for qPCR application by Springer et al., (2016) [27,34]. Further, the only commercially available kit for PCR-based Mucorales detection, MucorGenius® from PathoNostics, is assumed to have its roots in this assay [39]. Both qPCR assays, from Springer et al. and PathoNostics, were evaluated for clinical samples as listed in Table 2.

### 4.5. Pan-Fungal PCR Assays

Finally, Mucorales infections can also be detected using pan-fungal primers and identified by subsequent sequencing. Pan-fungal PCR assays have the advantage, that they detect any (at least in theory) fungal DNA, even from uncultured, rare, or unknown pathogenic fungi. Therefore, they can be applied if there is no clear suspicion of the pathogen involved. However, this non-specificity in detection also prevents direct identification of the fungus. Pan-fungal PCR assays are generally combined with Sanger sequencing of amplicons, which requires single-species PCR products and prolongs the time until diagnosis. To address this problem, Valero et al., (2016) developed a pan-fungal qPCR combining in the same PCR reaction: (i) a DNA-binding fluorescent dye for the detection of fungi in general, (ii) a multiplex application of group-specific fluorescent labelled-probes, and (iii) melt curve analysis [43]. This approach allowed group or species identification without sequencing in 78% or 44% of the PCR-positive samples, respectively, leading to faster diagnosis [43]. 

Pan-fungal PCR assays were tested in multiple studies, displaying a large range of sensitivities depending on the sample material and the primers used. As these studies were too diverse to be summarized in Table 2, they will be described in more detail here. Gade et al., (2017) compared three different PCR assays targeting the ITS, D1/D2, and extended 28S region and found that fungal DNA could be amplified in 58%, 34%, and 100% of FF-PET samples from patients with invasive fungal infections (including 29% mucormycosis cases), respectively [44]. Wagner et al., (2018) found that qPCR + sequencing of the 18S rRNA gene demonstrated higher sensitivity (98%) than PCR + sequencing of the ITS region (87%) in 233 clinical samples [45]. Zeller et al., (2017) adapted primers from White et al., (1990) and Khot et al., (2009) for qPCR and reached a sensitivity of 90% for 98 patients with invasive fungal infections (including 6% mucormycosis cases) [46,47,48]. When comparing specific PCRs for Mucorales and *Aspergillus*, respectively, with two broad-range PCRs, Springer et al., (2019) found higher sensitivities with the specific PCR, especially in mixed infections [49]. Most diagnostic laboratories use in-house assays for panfungal PCR. The only currently available CE/IVD certified assay that also targets fungal 18S rRNA is the SepsiTest™ from Molzym (Molzym Molecular Diagnostic, Bremen, Germany) [50].

### 4.6. Target Genes and Sequence Databases

In most cases, primers target the fungal ribosomal RNA gene, which comprises sequence encoding for the 18S rRNA, 5.8S rRNA, 28S rRNA (including D1/D2), and 5S rRNA as well as the internal transcribed spacers (ITS1 and ITS2,) and the intergenic sequences (IGS1 and IGS2) [51]. CLSI recommends the use of the ITS region for species identification for Mucorales because it demonstrates good differentiation between the genera, although the resolution of the taxonomy of *Lichtheimia* spp. is incomplete [52]. As an alternative DNA target, they recommend the D1/D2 region [52]. Schwarz et al., (2006) investigated the similarities and variabilities in the ITS1-5.8S-ITS2 region of Mucorales [53]. They found high sequence similarities within the species (>98%) and much lower similarities between the species, which is the prerequisite for a genetic target for species identification [53]. In contrast, Nilsson et al., (2008) demonstrated that intraspecific variability within the former fungal group Zygomycota (comprised of the current taxa Mucoromycota and Zoopagomycota) is on average 3.24% in the ITS1-5.8S-ITS2 region, varying largely between species [54]. Therefore, it is difficult to set a general valid threshold of sequence similarity for species discrimination. The original collection of primers targeting the ITS region was published in 1990 by White et al. [47], comprising the primers ITS1 to ITS5, which are still used [26,45,55]. Further primers designed since then are summarized in Khot et al., (2009) [48], who themselves added 27 new broad-range primers to the list. 

However, some taxa cannot be sufficiently identified based on the ITS region only and need additional barcodes, which are mostly based on housekeeping genes [56]. Therefore, in some cases, target genes other than ribosomal were tested for the diagnosis of Mucorales. Baldin et al., (2018) designed a PCR assay targeting the spore coating protein homolog encoding CotH genes, which are uniquely and universally present in Mucorales [14]. They evaluated their method using experimentally infected mice for plasma, urine, and bronchoalveolar lavages as well as urine from patients with proven mucormycosis and concluded that the CotH gene was a promising target for diagnostics in urine samples [14]. Further, Caramalho et al., (2019) tested the mitochondrial rnl gene, which encodes for the large subunit of the rRNA, for diagnostic purposes, using a qPCR + HRM assay [35]. The authors found a 100% rate of correct identification of culture isolates and a relatively high sensitivity for the detection of Mucorales in FFPE samples of 71%. Mitochondrial genes are more protected from degradation and are present in higher copy numbers than nuclear DNA, which makes them promising candidates for diagnostic assays [35]. Finally, the cytochrome b gene was targeted by a probe-based qPCR + HRM Mucorales-specific assay, which was evaluated for culture isolates, fresh tissue, and FFPE tissue, resulting in sensitivities of 100%, 100%, and 56%, respectively [36].

### 4.7. Potential and Limitations of Molecular Diagnostic Tools

Molecular diagnostics have advantages and disadvantages compared with histopathologic and microbiological methods. The main advantage is that PCR-based methods allow quicker and earlier diagnosis, which can lead to an early initiation of therapy and therefore lower mortality [4]. For example, Legrand et al., (2016) demonstrated reduced mortality in severely burned patients with invasive wound mycormycosis due to implementation of a systematic qPCR screening of plasma samples using the EPA assay, which led to earlier diagnosis [42]. Further, taxa identification based on DNA-sequences is more objective and requires less expertise than identification based on morphology [56]. In this context, a state-of-the-art taxonomy, which includes molecular phylogeny, is available for the important medical genera *Rhizpopus*, *Lichtheimia*, and *Apophyses* [57]. Only recently, Wagner et al., (2020) revised the species concept of the genus *Mucor* by combining a multi-locus analysis of seven genes with phenotypic characteristics, mating tests, and maximum growth temperatures [57].

The main disadvantages of PCR-based methods are the lack of standardization and clinical evaluation. The quality of a diagnostic method is defined by its analytic and more importantly diagnostic sensitivity and specificity (Table 2). These parameters depend on the applied method but also on the type of sample material. Due to the low incidence of invasive mucormycosis, large evaluations on clinical samples are rare. In contrast, the determination of the analytic sensitivity (Limit of Detection) and specificity is not restricted by incidences. Further, many evaluations lack a negative control group (e.g., patients with no mycormycosis or healthy people), making it impossible to calculate specificity values.

Despite these difficulties, the Fungal PCR Initiative (FPCRI, www.fpcri.eu), working group of the ISHAM, aims to include PCR diagnostics in the EORTC/MSG criteria for fungal infections, which they achieved for *Aspergillus* PCR; *Candida*, Mucorales, and *Pneumocystis* PCR must follow. In this context, an inter-laboratory study on two simulated serum panels (spiked with Mucorales DNA) was performed in 2017–2018, with 23 European laboratories participating [19]. The study evaluated the reproducibility of different DNA extraction and qPCR methods. The methods used by the laboratories included the genus-specific assay from the EPA, the Mucorales-specific qPCR from Springer et al., (2016) [27], the species-specific assay from Hrncirova et al., (2010) [34], and the commercial MucorGenius kit (Pathonostics). The assays were compared concerning the Cq (quantification cycle) during qPCR, which is inverse to the amount of target nucleic acid that can be detected. Therefore, low Cq values indicate low detection thresholds and a high sensitivity. In general, the assay of the EPA and MucorGenius demonstrated lower Cq values than the other assays. However, some assays were used much less than others, which limited statistical power. Within the laboratories using the EPA assay, the only technical parameter, which demonstrated a significant impact on the result, was the qPCR platform, with Rotor-Gene achieving the lowest Cq values. Overall, the study demonstrated very good concordance in results throughout the laboratories and methods used [19].

**Table 2:** Evaluations of the most tested assays. Ap, *Apophysomyces*; Am, *Actinomucor*; C, *Cunninghamella*; L, *Lichtheimia*; M, *Mucor*; Rp, *Rhizopus*; Rm, *Rhizomucor*; S, *Syncephalastrum*; ev, evaluated; ng, not given; na, not applicable; MM, mucomycosis; D, day (calculated from day of conventional diagnosis); FF-PET, formalin-fixed paraffin embedded tissue; BAL, bronchoalveolar-lavages; EORTC, European Organisation for Research and Treatment of Cancer; MSG, Mycoses Study Group.

Assay	Ev. in	Samples	Pos. Control Group	Neg. Control Group	Sensitivity	Specificity	Calculated on	Detected Taxa
Semi-nested PCR + sequencing (Bialek 2005)	[11]	FF-PET (*n* = 52)	MM (histopathology diagnosis)	aspergillosis (histopathology diagnosis)	59%	100%	samples = patients	Rp. spp., M. sp., Rm spp., L. sp.
	[58]	fresh tissue (*n* = 9)	proven MM (EORTC/MSG criteria)	ng	100%	ng	samples = patients	Rp. spp.
		FF-PET (*n* = 18)			56%			Rp. spp.
	[12]	FF-PET (*n* = 21)	MM (histopathology diagnosis)	aspergillosis/cryptococcosis/candiasis (histopathology diagnosis)	100%	100%	samples = patients	Rp. sp., Rm. spp., C. spp., L. sp.
	[59]	FF-PET (*n* = 27)	proven MM (EORTC/MSG criteria)	ng	81%	ng	samples = patients	Rp. spp., M. spp., C. spp., Rm. spp., and L. spp.
	[60]	FF-PET (*n* = 30 from 20 patients)	MM (histopathology diagnosis)	aspergillosis (histopathology diagnosis)	68%	100%	samples	no sequencing
	[61]	fresh tissue (*n* = 28)	MM (histopathology diagnosis)	ng	86%	ng	samples = patients	no sequencing
		serum (*n* = 28)			0%			
	[62]	fresh tissue (*n* = 56)	MM (histopathology diagnosis)	aspergillosis (histopathology diagnosis)	100%	100%	samples = patients	Rp. sp., Rm. spp., L. sp.
	[63]	serum (*n* = 62 from 31 patients)	MM diagnosis	no MM diagnosis	0%	100%	samples	na
	[32]	tissue samples (rhino-orbito-cerebral) (*n* = 50)	diagnosed rhino-orbito-cerebral-MM	ng	100%	ng	samples = patients	Rp. spp., Ap. sp.
qPCR +sequencing of 18S amplicon (Springer 2016a)	[27]	culture isolates (*n* = 28)	Ap. spp., *Cokeromyces* sp., C. spp., M. spp., Rm. sp., Rp. spp., *Saksenaea* sp., S. sp.	other clinically relevant fungi		100%	18S + 28S assay	na
		fresh (*n* = 3 from 3 patients) and FF-PET (*n* = 14 from 11 patients)	proven invasive MM	proven non-Mucorales IFD	90%	88%	18S + 28S assay	Rm. spp., L. spp., Rp. spp.
	[64]	FF-PET (*n* = 16 from 15 patients)	proven invasive MM (EORTC/MSG criteria)	patients without signs or symptoms typical for IFD	91%	100%	patients	Rm. spp., L. spp., Rp. spp.
					83%	100%	samples	
qPCR +sequencing of 18S amplicon (Springer 2016a) ff		serum (*n* = 52 of 5 patients)	Proven/probable invasive MM (EORTC/MSG criteria)	ng	100%	ng	patients	Rm. spp., L. spp., Rp. spp., M. spp., Am. sp.
					25%	ng	samples	
	[49]	FF-PET (*n* = 46)	MM (histopathology or broad range 18S PCR/sequencing diagnosis) (*n* = 2)	No MM (histopathology or broad range 18S PCR/sequencing diagnosis)	100%	93%	samples	Rp. spp.
	[65]	BAL (*n* = 99 from 96 patients)	proven/probable invasive fungal infection (MM: *n* = 6)	no invasive fungal infection	68%	93%	samples (supernatant + pellet)	no sequencing
qPCR (MucorGenius®, PathoNostics)	[66]	pulmonary samples *^1^ (*n* = 319 patients)	proven/probable MM (EORTC/MSG criteria) (*n* = 10)	proven/probable aspergillosis (EORTC/MSG criteria) (*n* = 63)	90%	98%	samples = patients	ng
	[39]	blood samples *^2^ (*n* = 106 from 16 patients)	proven/probable MM (EORTC/MSG criteria) (*n* = 10)	ng	75%	ng	patients	ng
					44%	ng	samples (D-20 to D75)	ng
	[19]	spiked serum (*n* = 28 samples in 4 labs)	spiked serum	not-spiked serum	84%	100%	samples	ng
qPCR (EPA/Bellanger 2018)	[67]	culture isolates (*n* = 19)	L. spp., Rp. spp., M. spp., Rm. spp.	other clinically relevant fungi	na	100%	isolates	na
		frozen serum (*n* = 51 from 10 patients)	proven MM	healthy/hematological malignancy/aspergillosis/pneumocystis infection	90%	100%	patients	L., Rm., M./Rp.
					51%	100%	samples (D-68 to D29 from time of diagnosis)	
	[13]	frozen serum (*n* = 194 from 44 patients)	proven MM (EORTC/MSG criteria)	ng	88%	ng	patients	L., Rm., M./Rp.
					81%	ng	samples (D-32 to D17 from time of diagnosis)	
	[66]	pulmonary samples *^1^ (*n* = 319 patients)	proven/probable MM (EORTC/MSG criteria) (*n* = 10)	proven/probable aspergillosis according to EORTC/MSG criteria (*n* = 63)	100%	96%	samples = patients	L., Rm., M./Rp., C.
	[68]	BAL (*n* = 450 from 374 patients)	proven/probable MM (EORTC/MSG criteria)	other or no fungal infections	100%	97%	patients	L., Rm., M./Rp.
	[42]	plasma (*n* = 418 from 77 patients)	proven/probable invasive wound MM (EORTC/MSG criteria)	ng	100%	ng	patients (earlier than standard diagnosis)	L., M./Rp.
	[19]	spiked serum (*n* = 112 samples in 16 labs)	spiked serum	not-spiked serum	90%	97%	samples	L., Rm.

*^1^ bronchoalveolar-lavages, tracheal aspirations, sputum, pleural fluids, or lung biopsies. *^2^ whole blood, serum, or plasma.

### 4.8. Future Perspectives and Outlook

Besides the ongoing need for large multi-center evaluation studies and standardization, there are also technical developments and improvements in molecular diagnostics. Costs for whole genome sequencing are currently dropping, allowing intensified usage in applied and basic clinical research [69,70,71,72] as well as outbreak management. For example, Garcia-Hermoso et al., (2018) demonstrated that an outbreak of *Mucor circinelloides* in a burn unit of a French hospital was caused by cross-transmissions between patients as well as contaminations with a heterogeneous pool of strains from an unknown environmental reservoir [72].

Further, although not yet applied to fungal infections, the CRISPR-Cas mechanism appears to be a promising method for future molecular diagnostics [73]. In nature, CRISPR-Cas is part of the antiviral defense of bacteria [74]. It involves a specific single-stranded nucleic acid and an enzyme with endonuclease activity [68]. Within the Cas-enzyme family, Cas9, Cas12, and Cas13 were adapted for diagnostic purposes so far, mostly for the detection of viral or human nucleic acids [75]. Currently, all systems, except the CRISPR-Chip device, rely on upstream (isothermal) amplification of the DNA/RNA [74]. The diagnostic tools are still in development and mostly aim for implementation as an instrument-free assay with a read-out via lateral-flow assay or fluorescence detection by Smartphone [74]. Compared to PCR-based techniques, it displays higher specificity while maintaining adequate sensitivity, and is a cheap and easy application, which indicates great potential for point-of-care applications and low infrastructure settings [74,75].

Finally, there are advances in other fields of Mucorales diagnostics, which were not in the main scope of this review but will be addressed here shortly. Burnha-Maurish et al., (2018) observed a monoclonal antibody (2DA6) to be highly reactive with purified fucomannan of *Mucor sp.* [76]. A constructed lateral flow immunoassay for detection of Mucorales demonstrated good results for bronchoalveolar lavages, serum, urine, and tissue specimens. A murine model supported the rapid and accurate detection of *Rhizopus delemar*, *Lichtheimia corymbifera*, *Mucor circinelloides,* and *Cunninghamella bertholletiae* [77]; however, more clinical data is needed. Further, Koshy et al., (2017) studied breath volatile metabolite profiles (thermal desorption gas chromatography/tandem mass spectrometry (GC–MS)), applying an experimental murine model of invasive mucormycosis including *Rhizopus arrhizus var. arrhizus*, *R. arrhizus var. delemar,* and *R. microsporus* [78]. The volatile metabolite sesquiterpene displayed distinct breath profiles and distinguished mucormycosis from aspergillosis. The metabolomic-breath test appears promising, but also needs further clinical evaluation. Another diagnostic approach is the evaluation of specific cytokine-profiles in response to a Mucorales infection [79]. The analysis of fungus-reactive T cells in the diagnostic management of infections due to Mucorales needs further evaluation, as only limited data are available. Low level concentration of mold-reactive T cells was present in healthy donors, compared with increased CD154+ T cells in patients with invasive fungal infections [80]. Beyond the lab: imaging provides a significant role in diagnosis of fungal disease; volumetric high-resolution computed tomography (CT) is the standard of care, although there is no radiologic pattern pathognomonic for infections of Mucorales; and the reversed halo and hypodense signs are typical for pulmonary diseases [80]. In non-neutropenic patients, CT imaging may present with atypical patterns [80]. 

## Figures and Tables

**Table 1:** Selection of molecular diagnostic assays for Mucorales. C, *Cunninghamella*; L, *Lichtheimia*; M, *Mucor*; Rp, *Rhizopus*; Rm, *Rhizomucor*; S, *Syncephalastrum*; Fl, fragment length; np, not published; (q)PCR, (quantitative) polymerase chain reaction; RFLP, restriction fragment length polymorphism; HRM, high resolution melt curve; ESI-MS, electrospray-ionization mass spectrometry; f, forward primer; r, reverse primer; p, probe; rRNA, ribosomal RNA; CotH, spore coat protein; rnl, large ribosomal RNA; ITS, internal transcribed spacers.

Type	Method	Target Gene	Primer Specificity	Identification Level	Primer/Probe	Fl (bp)
(q)PCR + sequencing	semi-nested PCR + sequencing (Bialek 2005) [11]	18S rRNA	Mucorales	species, Rm. only genus	f1−ATTACCATGAGCAAATCAGA, r1−TCCGTCAATTCCTTTAAGTTTC, f2 = f1, r2–CAATCCAAGAATTTCACCTCTAG	175–177
	Probe-based qPCR +sequencing of 18S amplicon (Springer 2016a) [27]	18S rRNA	Mucorales	genus	f−TTA CCRTGAGCAAATCAGARTG, r–AA TCYAAGAATTTCACCTCTAGCG, p–TYRR(G)G(G)B(A)T(T)T(G)T(A)TTT *^1^	175
		28S rRNA			f−TTTGGGAATGCAGCCT, r−TCARAGTTCTTTTCAWCTTTCCCT, p–CGARARACCGATAGCRAACAAGTACCGT	107
PCR + RFLP	semi-nested PCR + RFLP (Zaman 2017) [32]	18S rRNA	Mucorales	species, genus	See Bialek 2005 *^2^	175–177
	multiplex PCR + RFLP (Machouart 2006) [33]	18S rRNA	Rp sp., Rm sp., M. sp., and L. corymbifera	species, genus	f−TGATCTACGTGACAAATTCT + f−TGATCTACGCGAGCGAACAA + f−TGATCTACGTGACATATTCT + f−TGATCTACACGGCATCAAAT, r–AGTAGTTTGTCTTCGGKCAA *^3^	approx. 830
PCR + gel electrophoresis	PCR + electrophoresis (Baldin 2018) [14]	CotH	Mucorales	Mucorales	np	np
qPCR + HRM	nested-qPCR + HRM (Hrncirova 2010) [34]	18S rRNA	Mucorales	species, genus	See Bialek 2005	175–177
	qPCR + HRM (Caramalho 2019) [35]	mitochondrial rnl	Mucorales	genus/species	f−GGTGTAGAATACAAGGGAGTCGA, r−GGAGAAATCCGCCCCAGATAA	124
	FRET-qPCR + HRM (Hata 2008) [36]	cytochrome *b*	Mucorales	genus	f−TAGGAATTACAGCAAAT, r−CCAATGCAAACTCC, anchor-ACAATTTTCTTATTCTTCTTAGTATTAG, Donor–TTTATTCTTATTC	167
qPCR	Probe-based qPCR (EPA) [37]	n. g.	L.	genus	f−CACCGCCCGTCGCTAC, r−GCAAAGCGTTCCGAAGGACA, p−ATGGCACGAGCAAGCATTAGGGACG	118
			Rm.	genus	f−CACCGCCCGTCGCTAC, r−GTAGTTTGCCATAGTTCGGCTA, p–TGGCTATAGTGAGCATATGGGAGGCT	105
			M. and Rp.	2 genera	f-CACCGCCCGTCGCTAC, r−CCTAGTTTGCCATAGTTCTCA *^4^ GCAG, p–CCGATTGAATGGTTATAGTGAGCATATGGGATC	105
	Probe-based qPCR (Bellanger 2018) [38]	18S rRNA	C.	genus	f–TGTGGCTATGCAGCTGGTCA, r−ACACATTCAGGCACGAAGGC, p–TCGGTCGGCGTGGTTCTCTGCCCA	162
	MucorGenius®-qPCR (PathoNostics)	28S (according to [39])	Mucorales	order	according to [39], similar to Springer 2016a	np
Multiplex qPCR	2 × Multiplex qPCR (Salehi 2016) [40]	ITS 2	Quadriplex assay: Rp. microsporus, Rp. oryzae, M., and C. bertholletiae	genus/species	f−TGAATCATCRARTCTTTGAACGCA, r−ATATGCTTAAGTTCAGCGGGT, species-specific probes (see Salehi 2016)	approx. 300
			Triplex assay: L., S., and Rm.	genus/species	f−GAATCATCGARTTCTYGAACGCA, r−ATATGCTTAAGTTCAGCGGGT, species-specific probes (see Salehi 2016)	approx. 350
	Multiplex probe-based qPCR (Bernal-Martinez 2013) [41]	ITS 1	Rp. oryzae	species	f−TCTGGGGTAAGTGATTGC, r–GCGAGAACCAAGAGATCC, p–CGCGATAACCAGGAGTGGCATCGATCAAATCGCG	192
		ITS 1	Rp. microsporus	species	F–CTTCTCAGTATTGTTTGC, r−ATGGTATATGGTAAAGGG, p-CGCGATCCTCTGGCGATGAAGGTCGTATCGCG	187
		ITS 2	M.	genus	f−GTCTTTGAACGCAACTTG, r−CCTGATTTCAGATCAAAT, p–CGCGATTTCCAATGAGCACGCCTGTTATCGCG	263
PCR + microarray	Multiplex PCR + microarray (Spiess 2007) [29]	ITS 1	M. racemosus, Rp. microsporus, Rp. oryzae (and 12 other non-Mucorales fungi)	species	9 different f primer + 3 different r primer *^5^ (see Spiess 2007)	np
	PCR + microarray (Hsiao 2005) [30]	ITS 1/5.8S rRNA/ITS 2	L. corymbifera, C. spp., Rp. oryzae, Rm. pusillus (and 60 other non-Mucorales fungi)	species	f–TCCGTAGGTGAACCTGCGG, r–TCCTCCGCTTATTGATATG*^6^	640
PCR + ESI-MS	PCR + ESI-MS (Massire 2013) [28]	28S rRNA, 18S rRNA, mitochondrial 18S rRNA and cytB, tub, hpr	Fungi	genus/species	16 primer pairs, detection range from broad-range fungal to order level specificity	72–154

*^1^ parentheses indicate nucleotide with locked nucleic acid modification, primers modified from Bialek et al., (2005) [11]. *^2^ restriction enzymes: BsrD I, Afl II, Eco 0109I, and Hae II. *^3^ mixture of specific forward primers and a degenerated reverse primer; restriction enzymes: PpuMI, XhoII, BmgBI, AseI, CspCI, AflII, XmnI, and AclI. *^4^ the A at this position was replaced by a T in Millon et al., (2016) and Legrand et al., (2016) [13,42]. *^5^ r primers were Cy3 modified; array was extended by Boch et al., (2015) [31]. *^6^ r primer was digoxigenin modified.
